# FGF21-FGFR4 signaling in cardiac myocytes promotes concentric cardiac hypertrophy in mouse models of diabetes

**DOI:** 10.1038/s41598-022-11033-x

**Published:** 2022-05-05

**Authors:** Christopher Yanucil, Dominik Kentrup, Xueyi Li, Alexander Grabner, Karla Schramm, Eliana C. Martinez, Jinliang Li, Isaac Campos, Brian Czaya, Kylie Heitman, David Westbrook, Adam R. Wende, Alexis Sloan, Johanna M. Roche, Alessia Fornoni, Michael S. Kapiloff, Christian Faul

**Affiliations:** 1 Division of Nephrology, Department of Medicine, The University of Alabama at Birmingham, Tinsley Harrison Tower 611L, 1720 2nd Avenue South, Birmingham, AL 35294 USA; 2 Katz Family Drug Discovery Center and Division of Nephrology and Hypertension, Department of Medicine, Leonard M. Miller School of Medicine, University of Miami, Miami, FL USA; 3 Division of Nephrology and Hypertension, Center for Translational Metabolism and Health, Feinberg Cardiovascular and Renal Research Institute, Northwestern University, Chicago, IL USA; 4 Departments of Ophthalmology and Medicine, Stanford Cardiovascular Institute, Stanford University, 1651 Page Mill Road, Mail Code 5356, Palo Alto, CA USA; 5 Division of Nephrology, Department of Medicine, Duke University School of Medicine, Durham, NC USA; 6 Department of Pediatrics and Interdisciplinary Stem Cell Institute, Leonard M. Miller School of Medicine, University of Miami, FL Miami, USA; 7 Division of Molecular & Cellular Pathology, Department of Pathology, The University of Alabama at Birmingham, Birmingham, AL USA

**Keywords:** Cell biology, Cell signalling, Growth factor signalling, Cardiology, Cardiovascular biology, Cardiac hypertrophy, Endocrinology, Endocrine system and metabolic diseases, Diabetes, Diabetes complications

## Abstract

Fibroblast growth factor (FGF) 21, a hormone that increases insulin sensitivity, has shown promise as a therapeutic agent to improve metabolic dysregulation. Here we report that FGF21 directly targets cardiac myocytes by binding β-klotho and FGF receptor (FGFR) 4. In combination with high glucose, FGF21 induces cardiac myocyte growth in width mediated by extracellular signal-regulated kinase 1/2 (ERK1/2) signaling. While short-term FGF21 elevation can be cardio-protective, we find that in type 2 diabetes (T2D) in mice, where serum FGF21 levels are elevated, FGFR4 activation induces concentric cardiac hypertrophy. As T2D patients are at risk for heart failure with preserved ejection fraction (HFpEF), we propose that induction of concentric hypertrophy by elevated FGF21-FGFR4 signaling may constitute a novel mechanism promoting T2D-associated HFpEF such that FGFR4 blockade might serve as a cardio-protective therapy in T2D. In addition, potential adverse cardiac effects of FGF21 mimetics currently in clinical trials should be investigated.

## Introduction

The global prevalence of type 2 diabetes (T2D) is growing rapidly and is expected to reach 300 million by 2025. Diabetes is a risk factor for congestive heart failure, and cardiovascular complications are the leading cause of diabetes-related morbidity and mortality^1^. Diabetic cardiomyopathy is termed as heart failure in the absence of other comorbid conditions and is associated with both diastolic and systolic cardiac dysfunction, as well as pathological cardiac myocyte hypertrophy^1^. Cardiac remodeling includes defects in calcium handling and myocyte contractility, altered myocyte metabolism, and an increase in oxidative stress and mitochondrial dysfunction, as well as induction of interstitial myocardial fibrosis. Over 40% of T2D patients have increased left ventricular (LV) mass and wall thickness, conferring additional risk of heart failure that is independent of the other features of metabolic syndrome (hypertension, hyperlipidemia, and obesity) often present in these patients^2–4^. Importantly, hyperglycemia and the associated metabolic abnormalities in T2D are considered major factors promoting heart failure with preserved ejection fraction (HFpEF)^5^. An area of intense current research, HFpEF represents about half of all patients with heart failure and is a syndrome for which therapeutic options are limited to the recent discovery of the efficacy of sodium-glucose cotransporter-2 (SGLT2) inhibitors^6^. Here we consider the potential role of FGF21 in the development of pathological concentric cardiac hypertrophy and diabetic cardiomyopathy.

The fibroblast growth factor (FGF) family consists of 22 members that by activation of FGF receptor (FGFR) tyrosine kinases regulate cellular proliferation, survival, migration and differentiation^7^. FGF19, FGF21 and FGF23 are distinguished by their low heparin binding affinity and their function as hormones acting on distant target organs^8^. As the endocrine FGFs bind heparin poorly, cellular activation requires klotho, a family of single-pass transmembrane proteins (α- and β-klotho) that serve as co-receptors to facilitate FGF-FGFR binding. The klotho co-receptors have a more restricted expression pattern than FGFRs, thereby conferring tissue-specificity to the actions of endocrine FGFs^8^.

Produced mainly by hepatocytes, FGF21 functions as a major regulator of metabolism and energy homeostasis during fasting conditions^7,9^. For example, by binding FGFR1/β-klotho receptors in adipocytes, FGF21 induces glucose uptake and fatty acid storage^10^. Accordingly, pharmacological administration of FGF21 can have beneficial effects in animal models, including a reduction in blood glucose, triglyceride and cholesterol levels, weight loss, and increased life span^10^. In patients with T2D and obesity, however, markedly elevated serum FGF21 levels have been observed^11–14^, prompting the hypothesis that obesity is a FGF21-resistant metabolic state^15^. Elevated serum FGF21 levels are also present in hypertension^16^, dilated cardiomyopathy^17^, coronary artery disease^18–20^, acute myocardial infarction^21^, and atrial fibrillation^22^. Notably, FGF21 expression has been found to be increased in HFpEF patients with diastolic dysfunction^23–25^. FGF21 appears to have direct effects on the heart, including cardio-protective actions of short-term FGF21 elevations during ischemia/reperfusion injury, β-adrenergic activation, or hypertension^26–35^.

Concentric cardiac hypertrophy and diastolic dysfunction are prominent features of diabetic cardiomyopathy and HFpEF^5^. Concentric cardiac hypertrophy is characterized by an increase in relative wall thickness (left ventricular wall thickness to internal diameter ratio) that is primarily a reflection of increased cardiac myocyte width, while, in contrast, eccentric cardiac hypertrophy is characterized by decreased relative wall thickness and relative myocyte lengthening. Concentric hypertrophy can be induced by activation of an extracellular signal-regulated kinase 1/2 (ERK1/2)—p90 ribosomal S6 kinase type 3 (RSK3)—serum response factor (SRF)-dependent gene expression regulatory pathway^36–38^. It has been observed that in cardiac myocytes FGF21 activates ERK1/2 and, like ERK1/2 gene deletion, unstressed FGF21 knock-out mice exhibit eccentric cardiac hypertrophy^26^. Here we consider the novel hypothesis that, despite its ability to promote myocyte survival, the elevation of FGF21 in T2D is not beneficial, but deleterious, such that in the context of T2D elevated FGF21 promotes pathological concentric cardiac hypertrophy. Studies performed both in vitro and in vivo suggest that FGF21 is a key humoral mediator of the pathological remodeling in diabetic cardiomyopathy. FGF21-FGFR4 signaling might constitute a novel therapeutic target for heart failure associated with T2D.

## Results

### FGF21 induces concentric cardiac hypertrophy in mice

**Figure 1:**
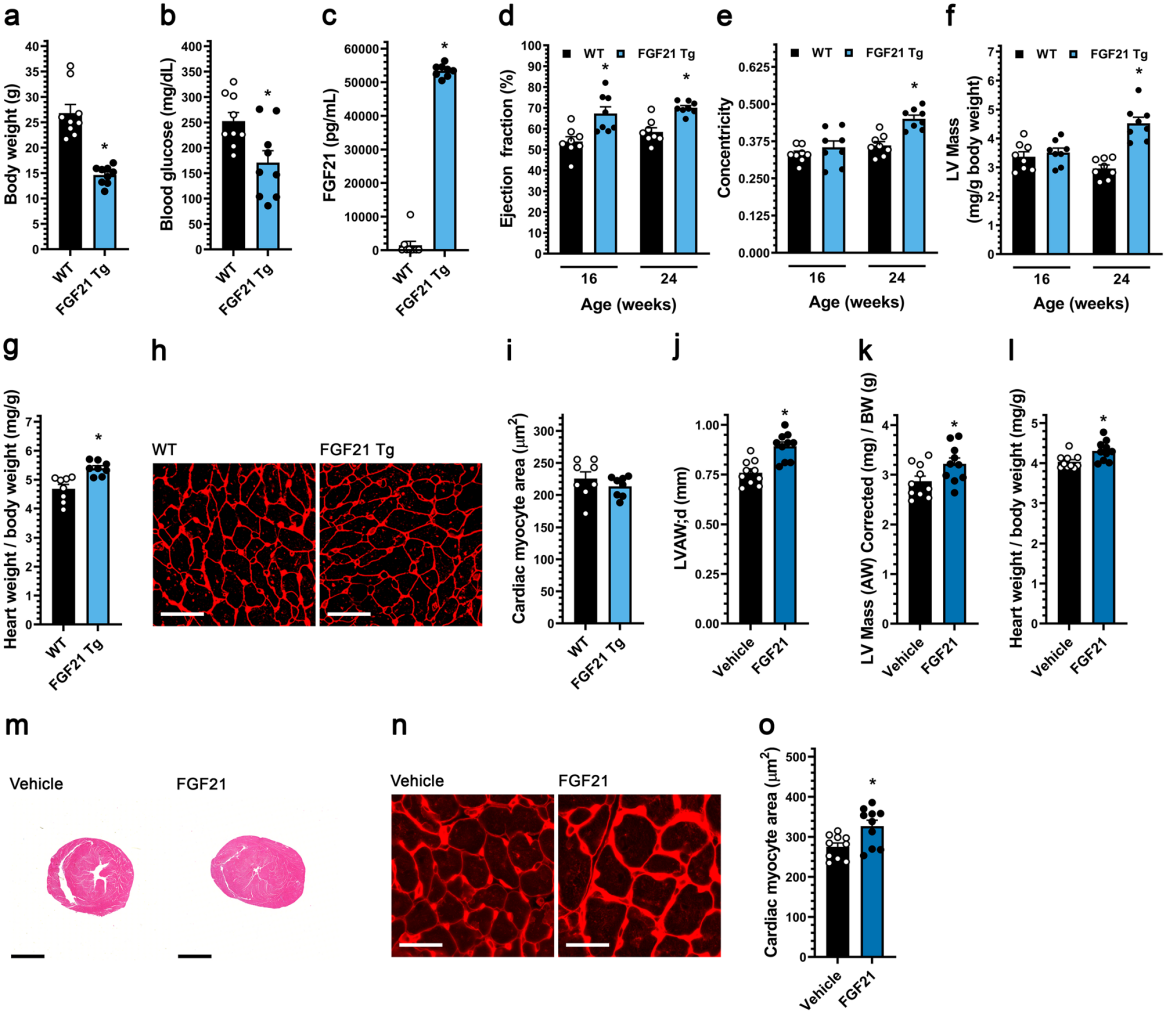
Systemic FGF21 elevation in mice induces concentric cardiac hypertrophy. (**a**–**i**) Analysis of FGF21 transgenic (Tg) mice and wild-type littermates at 24 weeks of age unless otherwise indicated. (**a**) Body weight, (**b**) blood glucose levels, and (**c**) serum FGF21 levels. (**d**) Ejection fraction, (**e**) concentricity [relative wall thickness = (LVAW;d + LVPW;d)/LVID;d], and (**f**) left ventricular (LV) mass, as determined by echocardiography at 16 and 24 weeks of age. (**g**) Heart weight/body weight ratio, (**h**, **i**) wheat germ agglutinin staining of LV tissue (scale bar = 25 µm) and cardiac myocyte cross-section area. (**j**–**o**) Wildtype mice were injected i.v. with FGF21 or vehicle for five consecutive days before analysis on the 6th day. (**j**) LV anterior wall thickness (diastole), and (**k**) LV mass/body weight ratio, as determined by echocardiography. (**l**) Gravimetric heart weight/body weight ratio. (**m**) Hematoxylin and eosin-stained transverse heart sections (scale bar = 2 mm). (**n**, **o**) Wheat germ agglutinin staining of LV tissue (scale bar = 25 µm) and cardiac myocyte cross-section area. Statistical significance was determined by 2-way ANOVA with post-hoc testing with Sidak's multiple comparisons test (**d**–**f**), or by two-tailed t-test. All values are expressed as mean ± SEM. (**a**, **b**) N = 9; (**c**) N = 8–9; (**d**–**f**) N = 8, *p ≤ 0.05 vs. WT of same age; (**g**, **i**) N = 8, *p ≤ 0.05 vs. WT; (**j**–**l**, **o**), N = 10, *p ≤ 0.05 vs. vehicle. For the complete set of echocardiography parameters see Supplementary Tables 1 and 3.

To test for possible effects on cardiac structure and function by elevated FGF21 levels, a FGF21 transgenic mouse (FGF21-Tg) was utilized in which FGF21 is systemically elevated by expression in the liver under the control of the apolipoprotein E (ApoE) promoter^39^. Consistent with the original report^39^, at 24 weeks of age these mice had reduced body weight and blood glucose levels, and a 200-fold increase in serum FGF21 levels (Fig. 1a–c). While the analysis of cardiac function in FGF21-Tg mice was confounded by their significantly reduced body weight and apparent failure-to-thrive, by echocardiography these mice exhibited a persistent elevation in ejection fraction and relative LV hypertrophy, as indicated by increased LV mass to body weight ratio and relative wall thickness (“concentricity index”)^40^, consistent with the development of concentric cardiac hypertrophy (Fig. 1d–f; Supplementary Fig. 1a; Supplementary Table 1). Relative cardiac hypertrophy in FGF21-Tg mice was confirmed by gravimetric measurement of heart weight to body weight ratio (Fig. 1g). Paradoxically, cross-sectional area of cardiac myocyte was not increased in FGF21-Tg mice, presumably lower in proportion to the decreased body weight (Fig. 1h,i). Histological and gene expression analyses did not show evidence of cardiac fibrosis in FGF21-Tg mice (Supplementary Fig. 2a–d). Taken together, overexpression of exogenous FGF21 in mice appeared to induce a relative concentric cardiac hypertrophy, although the decreased body weight of the transgenic mice precluded definitive conclusions about the cardiac phenotype.

To determine if FGF21 elevation could induce concentric cardiac hypertrophy in the absence of confounding effects on body weight, adult wildtype BALB/cJ mice were injected intravenously (i.v.) twice daily with 40 μg/kg of recombinant FGF21 protein or isotonic saline for five days, followed by echocardiography and tissue collection on the sixth day. Echocardiographic analysis revealed that FGF21 increased LV anterior wall thickness, LV mass and concentricity index (Fig. 1j,k; Supplementary Fig. 1b; Supplementary Table 2). Accordingly, gravimetric and histological analysis showed an elevation in indexed heart weight (Fig. 1l,m) and in the cross-sectional area of individual cardiac myocytes (Fig. 1n,o). These results demonstrate that elevated FGF21 can rapidly induce concentric cardiac hypertrophy in wildtype mice.

### FGF21 promotes the concentric hypertrophy of adult rat ventricular myocytes in vitro

**Figure 2:**
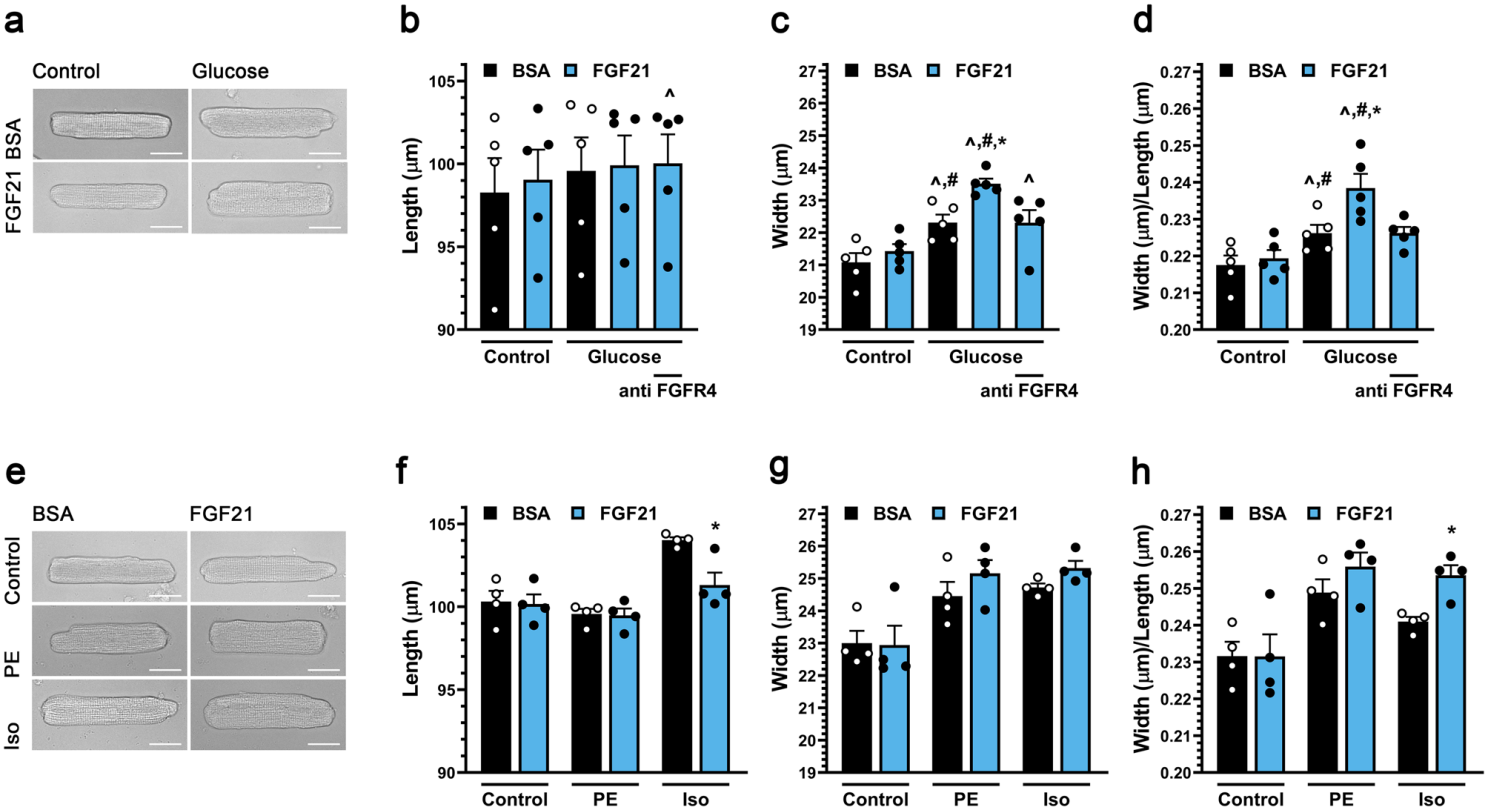
FGF21 induces hypertrophic growth of cultured cardiac myocytes in the presence of high glucose via FGFR4. (**a**) Transmitted light images of primary adult rat ventricular myocytes (ARVMs) treated with BSA (control), and mouse recombinant FGF21 (25 ng/ml) with or without 10 mM increased glucose (final 5.6 or 15.6 mM) for 48 h (scale bar = 25 µm), and (**b**–**d**) myocyte length, width, and width to length ratio. FGFR4-specific blocking antibody (anti-FGFR4; 10 mg/ml) was included as indicated. (**e**) Images of ARVMs treated with BSA (control), and FGF21 (25 ng/ml) with or without phenylephrine (PE; 20 µM) or isoproterenol (Iso; 10 µM) for 48 h (scale bar = 25 µm), and (**f**–**h**) myocyte length, width, and width to length ratio. Comparison between groups was performed in form of a one-way (**b**–**d**, **f**–**h**) ANOVA followed by post-hoc Tukey test. All values are expressed as mean ± SEM. (**b**–**d**) N = 5, ^**^**^p ≤ 0.05 vs. BSA Control, ^#^p ≤ 0.05 vs. FGF21 Control, *p ≤ 0.05 vs. Glucose Control. (**f**–**h**) N = 4, *p ≤ 0.05 vs. respective Control.

Systemic FGF21 elevation in vivo resulted in concentric cardiac hypertrophy. To demonstrate that FGF21 can induce cardiac myocyte hypertrophy in a cell autonomous manner, primary adult rat ventricular myocytes (ARVM) were studied in vitro. Stimulation by 25 ng/ml FGF2, an FGF isoform known to induce myocyte hypertrophy^41^, but not stimulation by FGF21 induced the hypertrophy of myocytes cultured in minimal medium (data not shown). Given that elevated FGF21 serum levels are associated with diabetic cardiomyopathy^8^, we considered that FGF21 might induce hypertrophy only in the presence of other stimuli, such as elevated glucose levels. Myocytes were cultured in the presence of 15.6 mM glucose, mimicking hyperglycemia of a serum level of 279 mg/dL and threefold the glucose concentration in standard minimal medium. While 48 h of FGF21 stimulation did not induce myocyte hypertrophy, culture in medium containing elevated glucose significantly increased myocyte width by 6%, while not affecting myocyte length, resulting in a significantly increased width:length ratio indicative of a mild “concentric” myocyte hypertrophy (Fig. 2a–d). Remarkably, addition of FGF21 to medium containing high glucose further increased myocyte growth in width without affecting myocyte length, such that ARVM cultured in FGF21 and 15.6 mM glucose were 12% wider and had a width:length ratio 10% greater than control cells.

We next considered that FGF21 might drive concentric remodeling in the presence of other stress stimuli. The α-adrenergic agonist phenylephrine induces a selective growth in width of cultured ARVM, while the β-adrenergic agonist isoproterenol induces growth in both width and length, resulting in a more symmetric ARVM hypertrophy^38^. Co-treatment with FGF21 did not significantly further increase the prominent growth in width induced by phenylephrine or isoproterenol (Fig. 2e–h). Remarkably, however, co-treatment with FGF21 inhibited the growth in length induced by isoproterenol, resulting in an increased width:length ratio comparable to that induced by phenylephrine and FGF21. These findings suggest that in the presence of hypertrophic stimuli, including elevated glucose levels, FGF21 will promote a “concentric” phenotype characterized by preferential growth in width of the cardiac myocytes.

**Figure 3:**
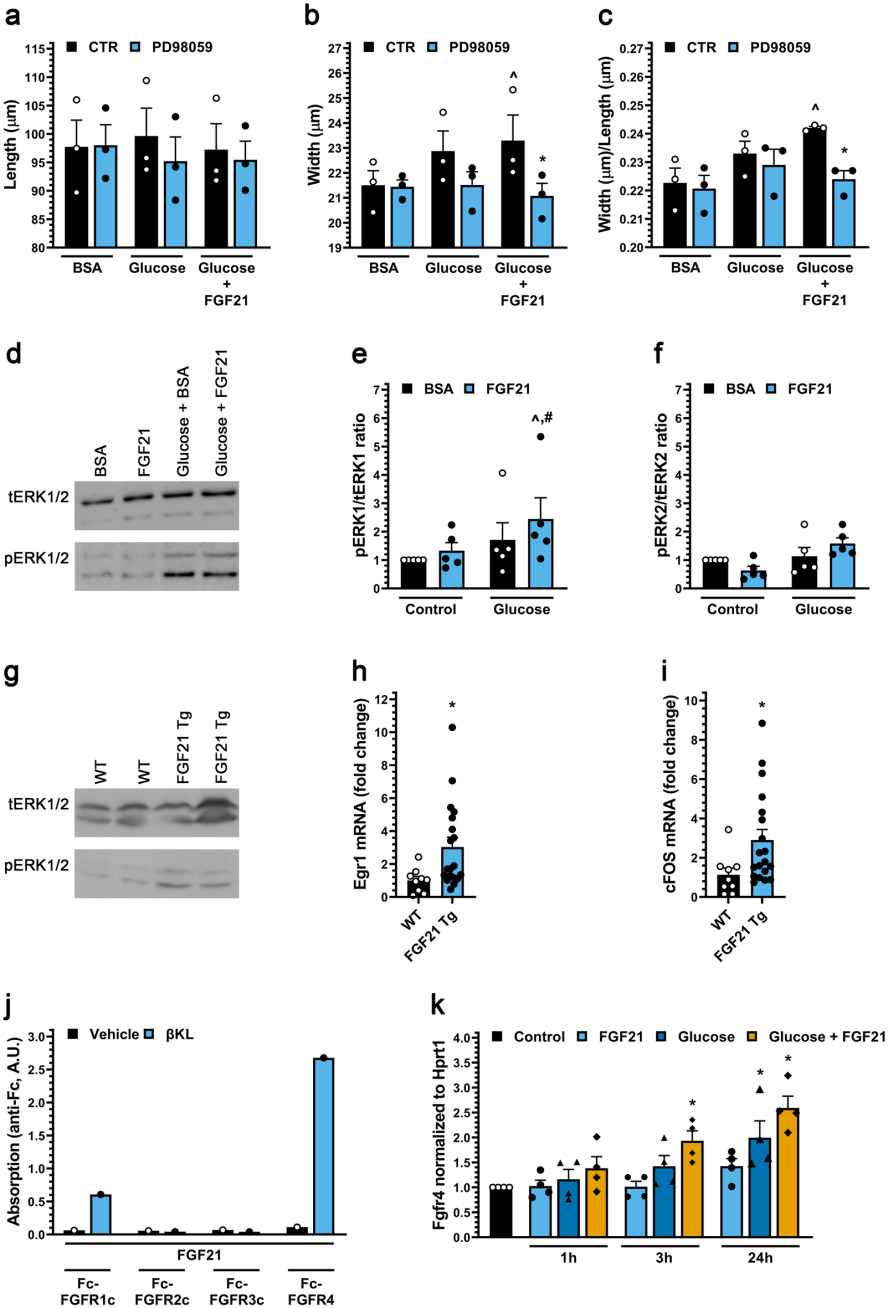
FGF21 activates ERK1/2 in cultured cardiac myocytes in the presence of high glucose and in heart tissue of mice. (**a**–**c**) Length, width and width to length ratio for ARVMs treated with BSA (control), FGF21 (25 ng/ml), increased glucose and/or the MEK inhibitor PD98059 (20 µM) for 48 h. Bars and colored symbols indicate average mean and means of independent experiments using different myocyte preparations, respectively. (**d**–**f**) Western blot analysis of ARVMs treated with BSA (control) or mouse recombinant FGF21 (25 ng/ml) with or without 10 mM glucose for 6 h. ERK1 is p44 and ERK2 is p42. (**g**) Analysis of cardiac tissue from FGF21 Tg mice and wild-type littermates at 8–12 weeks of age by Western blotting. (**h**, **i**) qRT-PCR for *Egr-1* and *c-Fos* mRNA using total RNA from heart tissue of FGF21 Tg mice and wild-type littermates at 8–12 weeks of age. (**j**) Binding of 1 µg of soluble β-klotho (βKL) or PBS, 500 ng of Fc-tagged FGFR 1c, 2c, 3c, or 4 to 96-well plates coated with 200 ng of FGF21. (**k**) qRT-PCR for *FGFR4* mRNA using total RNA isolated from ARVMs treated with BSA (control), FGF21 (25 ng/ml), and/or increased glucose (15.6 mM total). Comparison between groups was performed in form of a one-way (**a**–**c**, **k**) or two-way (**e**–**f**) ANOVA followed by post-hoc Tukey test or a two tailed t-test (**h**, **i**). All values are expressed as mean ± SEM. (**a**–**c**) N = 3, ^**^**^p ≤ 0.05 vs. BSA CTR, *p ≤ 0.05 vs. Glucose + FGF21 CTR; (**e**, **f**) N = 5, ^**^**^p ≤ 0.05 vs. BSA CTR, ^#^p ≤ 0.05 vs. FGF21 CTR; (**h, i**) N = 9−19, *p ≤ 0.05 vs. WT; (**k**) N = 4, *p ≤ 0.05 vs. CTR. All Western blots are cropped, and original blots are presented in Supplementary Fig. 3.

Concentric cardiac myocyte hypertrophy can be induced by activation of the ERK1/2 signaling pathway^36^. Addition of the MEK inhibitor PD98059 significantly inhibited the growth in width and increased width:length ratio of myocytes cultured in the presence of both FGF21 and high glucose (Fig. 3a–c). Western blot analyses revealed that co-treatment of ARVMs with FGF21 and glucose significantly increased the levels of phosphorylated ERK1 (Fig. 3d–f). Similarly, cardiac tissue from FGF21-Tg mice showed increased levels of phosphorylated ERK1/2 (Fig. 3g), as well as elevated mRNA levels of *Egr-1* and *c-Fos* (Fig. 3h,i) which are downstream targets of ERK1/2 signaling. ERK1/2 signaling is induced by FGF21 in cells expressing β-klotho and FGFRs^9^, and β-klotho (*Klb*) expression has been detected in ARVMs (Supplementary Table 3 and ^30^). In a binding assay using recombinant proteins, we found that in the presence of β-klotho, FGF21 bound the Fc-coupled ectodomain of FGFR4 preferentially to FGFR1c, while FGFR2c and FGFR3c did not detectably bind FGF21 at all (Fig. 3j). Interestingly, *Fgfr4* expression in ARVM was induced synergistically by high glucose and FGF21 (Fig. 3k), providing a potential mechanism for the permissive effects of high glucose on FGF21 signaling. Importantly, using a blocking antibody for FGFR4^42^, the synergy between glucose and FGF21 in promoting adult myocyte growth in width was found to require FGFR4 activation (Fig. 2b–d).

### Diabetic BTBR ob/ob mice develop FGFR-dependent cardiac hypertrophy

**Figure 4:**
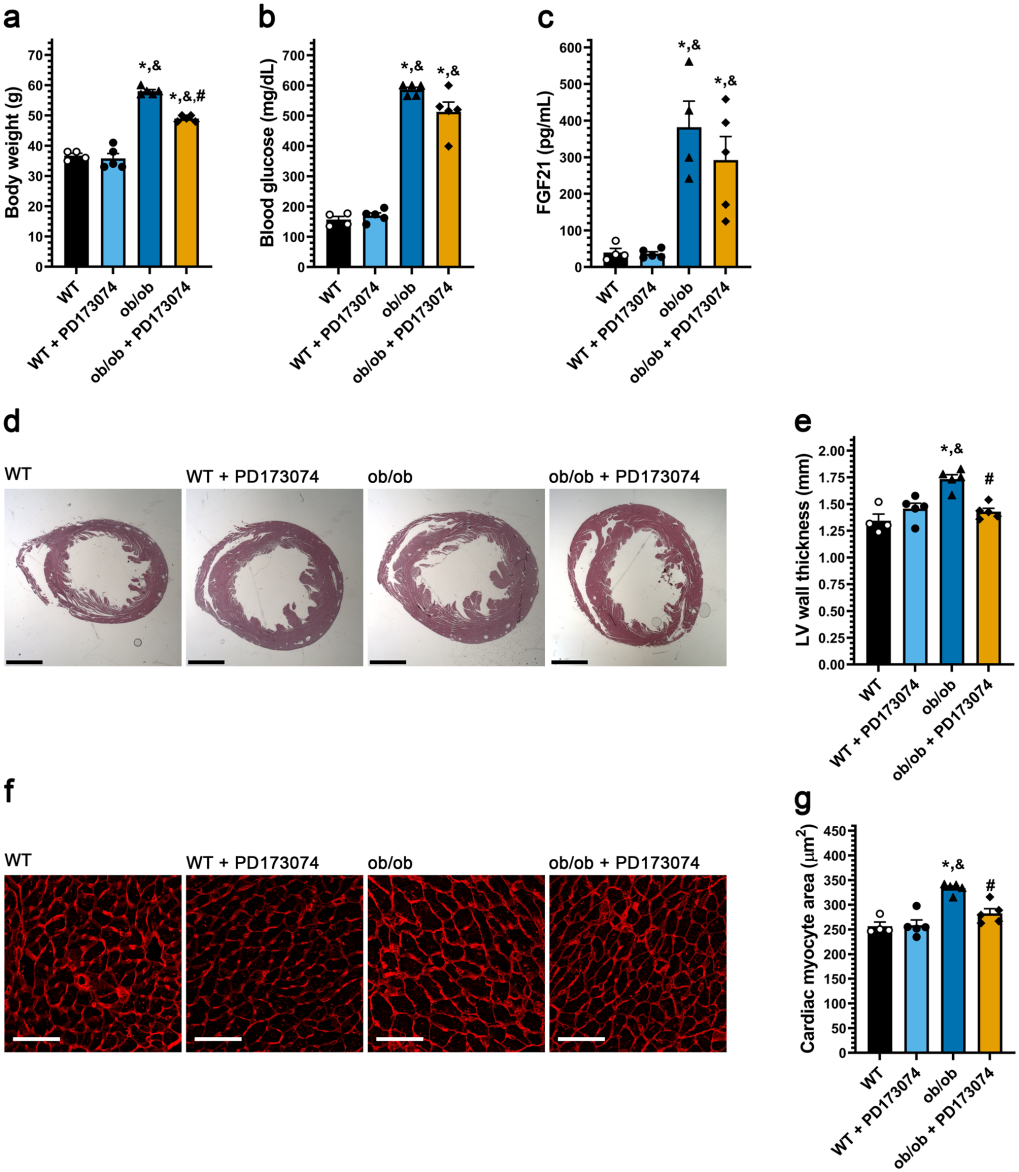
Systemic pan-FGFR blockade protects diabetic mice from cardiac hypertrophy. Three-month old ob/ob and wildtype mice were injected daily with 1 mg/kg PD173074 or vehicle solution for six weeks followed by endpoint analysis. (**a**) Body weight, (**b**) blood glucose levels, and (**c**) serum FGF21 levels. (**d**) Representative images of H&E-stained transverse heart sections (scale bar = 2 mm) and (**e**) quantification of the thickness of the LV wall in these images. (**f**) Wheat germ agglutinin-stained LV tissue (scale bar = 25 µm), and (**g**) quantification of cardiac myocyte area. Comparison between groups was performed in form of a one-way ANOVA followed by a post-hoc Tukey test (**a**–**c**, **e**, **g**). All values are expressed as mean ± SEM. (**a**–**c**, **e**, **g**) N = 4–5, *p ≤ 0.05 vs. WT, ^&^p ≤ 0.05 vs. WT + PD173074 of same age, ^#^p ≤ 0.05 vs. ob/ob of same age.

As high serum FGF21 levels are present in diabetic cardiomyopathy^8^ and FGF21 and high glucose directly promoted cardiac myocyte growth in width, we considered that FGF21/FGFR signaling might promote cardiac hypertrophy in this disease state. The genetic ob/ob mouse model lacking leptin is grossly obese and develops elevated blood glucose^43,44^ and FGF21 levels^14,45^, while exhibiting cardiac hypertrophy at six months of age^46^. ob/ob mice in the BTBR genetic background, which develop severe diabetes^47^, were treated at three months of age with the pan-FGFR inhibitor PD173074 by daily intraperitoneal (1 mg/kg i.p.) injection for six weeks. At endpoint, the elevated body weight and blood glucose levels in ob/ob mice^47,48^ were modestly reduced by PD173074 treatment, reaching significance only for body weight (Fig. 4a,b). Similarly, serum FGF21 levels were increased 15-fold in BTBR ob/ob mice compared to wild-type littermates and not significantly altered by PD173074 treatment (Fig. 4c). BTBR ob/ob mice developed cardiac hypertrophy without fibrosis (Supplementary Fig. 2e,f), as indicated by increased LV wall thickness (Fig. 4d,e) and cardiac myocyte cross-sectional area (Fig. 4f,g). Notably, the ob/ob-associated hypertrophy was inhibited significantly by PD173074 treatment. These results show that inhibition of FGFR signaling can diminish the development of cardiac hypertrophy despite minimally affecting hyperglycemia and obesity.

### FGFR4 blockade attenuates cardiac hypertrophy in diabetic db/db mice

**Figure 5:**
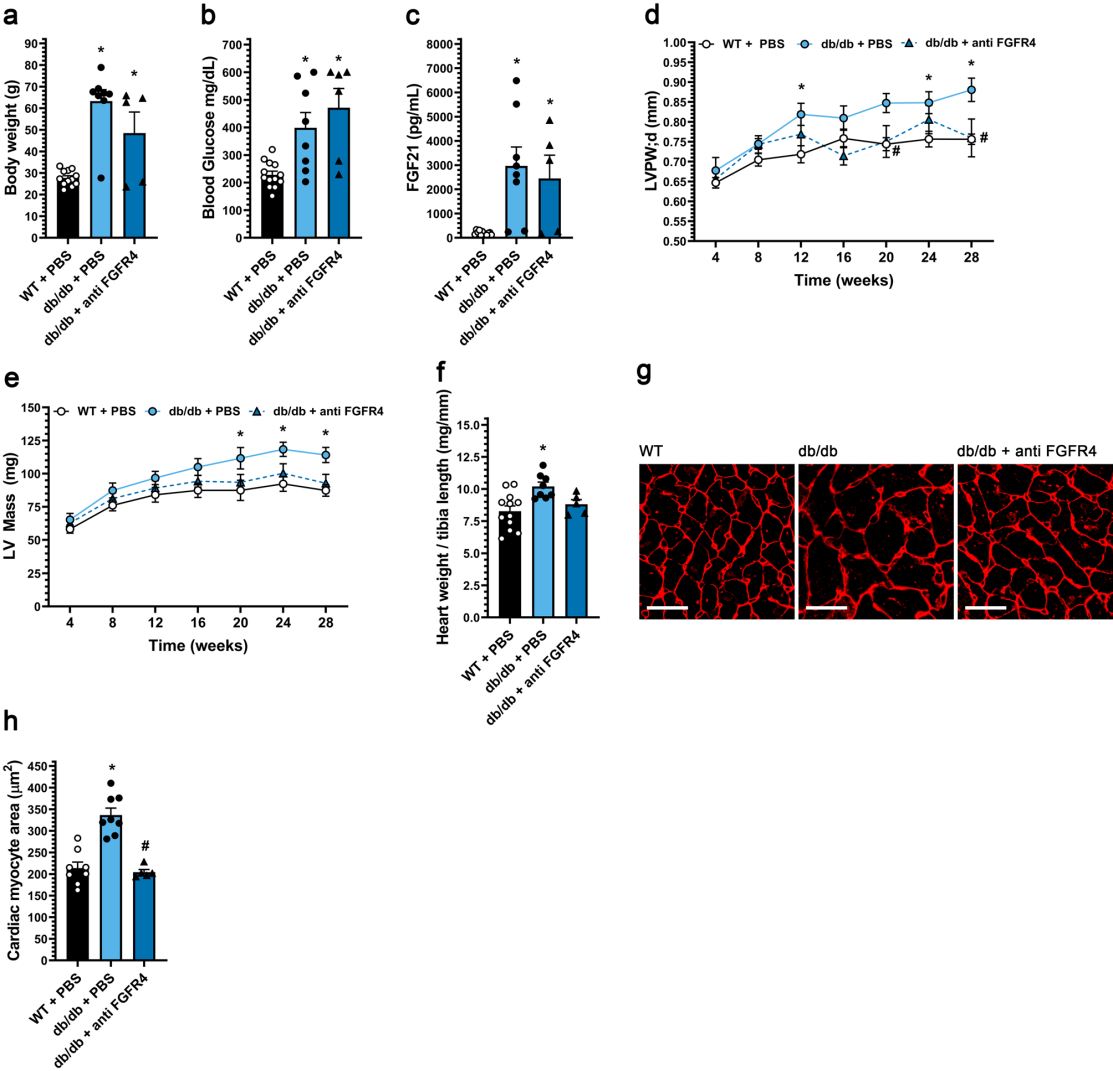
FGFR4 blockade protects diabetic mice from pathologic cardiac remodeling. db/db mice and wild-type (WT) littermates were treated for 24 weeks with either FGFR4 blocking antibody (25 mg/kg) or vehicle (PBS) on a bi-weekly basis, starting at 4 weeks of age. (**a**) Body weight, (**b**) blood glucose levels, and (**c**) serum FGF21 levels. (**d**) left ventricular (LV) posterior wall thickness in diastole, and (**e**) LV mass from 4 weeks until 28 weeks of age, as determined by serial echocardiography. (**f**) Gravimetric heart weight/tibia length ratio, (**g**) wheat germ agglutinin-stained LV tissue section (scale bar = 25 µm), and (**h**) cardiac myocyte cross-section area. Comparison between groups was performed in form of a one-way ANOVA (**a**–**c**, **f**, **h**) or a 2-way ANOVA (**d**, **e**) followed by a post-hoc Tukey test. All values are expressed as mean ± SEM. (**a**, **c**, **f**) N = 5–12; (**b**) N = 6–13; (**h**) N = 5–9, *p ≤ 0.05 vs. WT, ^#^p ≤ 0.05 vs. db/db. (**d**, **e**) N = 6–13, *p ≤ 0.05 vs. WT of same age, ^#^p ≤ 0.05 vs. db/db of same age. For the complete set of echocardiography parameters see Supplementary Table 3.

Our in vitro data suggest that FGFR4 is the primary FGFR isoform transducing hypertrophic FGF21 signals. This hypothesis was tested in vivo using the FGFR4 blocking antibody, in this case using db/db mice^49^, which lack the leptin receptor and develop a more severe cardiomyopathy that includes interstitial myocardial fibrosis. db/db mice, whether saline-injected controls or injected i.p. bi-weekly with FGFR4 blocking antibody (25 mg/kg) for 24 weeks starting at four weeks of age, exhibited elevated body weight, blood glucose and serum FGF21 levels (Fig. 5a–c). Notably, the progressive increase in LV wall thickness and LV mass in db/db mice was prevented by anti-FGFR4 treatment (Fig. 5d,e; Supplementary Fig. 1c). In addition, the increase in concentricity index at endpoint in db/db mice (Supplementary Table 3), indicative of concentric cardiac hypertrophy, was prevented by treatment with the FGFR4 antibody. Gravimetric analysis confirmed that the blocking antibody inhibited db/db-induced cardiac hypertrophy (Fig. 5f). Histologically, db/db mice had increased cardiac myocyte cross-sectional area (Fig. 5g,h), as well as interstitial myocardial fibrosis (Supplementary Fig. 2 g–j), that were both attenuated by the FGFR4 antibody. These results demonstrate that in diabetic db/db mice, FGFR4 activation promotes pathological cardiac remodeling, including concentric hypertrophy and interstitial fibrosis, providing further evidence for a critical role for FGF21-FGFR4 signaling in the cardiomyopathy associated with T2D.

## Discussion

The development of concentric cardiac hypertrophy is a key feature of the cardiomyopathy associated with diabetes and HFpEF^5^. Here we provide evidence that elevation in systemic FGF21 levels and the associated cardiac myocyte signaling constitutes a previously unrecognized mechanism for the induction of pathological concentric remodeling, thereby defining a new candidate target for therapeutic intervention in T2D. Blockade of FGFR signaling using both a pan-FGFR small chemical inhibitor and a FGFR4-specific blocking antibody inhibited the concentric hypertrophy associated with diabetic cardiomyopathy in mice. In addition, inhibition of FGFR4 signaling using the blocking antibody and a MEK inhibitor prevented the selective growth in width of adult myocytes in vitro induced synergistically by high glucose concentrations and FGF21. Thus, both gain and loss of FGF21-FGFR4 signaling in mouse models, as well as in vitro studies using adult cardiac myocytes, suggest that elevated FGF21 signaling promotes pathological concentric cardiac hypertrophy. Taken in the context of previously published human studies showing elevated FGF21 levels in patients with metabolic syndrome and HFpEF^9,23,24^, we proposed that FGF21 signaling is a conserved critical mechanism contributing to pathological concentric hypertrophy and heart failure.

We found that FGF21 induces myocyte hypertrophy via FGFR4. While others have shown that FGF21 could bind FGFR4-β-Klotho heterodimers^50^, as we show here, it has been suggested that FGF21 cannot activate downstream signaling via FGFR4^51,52^. Instead, we found that in adult cardiac myocytes, induction of hypertrophy by FGF21 could be inhibited by a FGFR4-specific blocking antibody. FGF21 is not the only member of the FGF family that can induce cardiac hypertrophy via ERK1/2 signaling. For example, presumably through activation of FGFR1, the paracrine factor FGF2 promotes cardiac hypertrophy under conditions of excess catecholamine stimulation and renin-angiotensin system activation^53^. In addition, we have shown that FGF23-FGFR4 signaling can induce cardiac hypertrophy in chronic kidney disease (CKD)^54,55^. However, of the endocrine FGFs, the association between elevated FGF21 systemic levels and diabetes and obesity is most well established^8^. Whether FGF23 is increased in human obesity is controversial^56^, and we found that FGF23 was not elevated in either the ob/ob or db/db mouse (data not shown). In addition, FGF19 levels are reduced in human obesity^8^. Thus, while FGF23 may be an important mediator of cardiac hypertrophy in CKD, FGF21 may have an especially important role in the induction of concentric hypertrophy in diabetic cardiomyopathy.

The identification of FGF21 as a mediator of pathological concentric hypertrophy stands in contrast to a literature purporting the beneficial effects of FGF21 on the cardiovascular system. While not excluding other mechanisms, including indirect systemic effects, many of these differences may be explained by ERK1/2-mediated differential regulation of concentric and eccentric cardiac hypertrophy and ERK1/2-mediated cardioprotection^36,57^. As observed in vitro for isoproterenol-treated myocytes, FGF21 may promote concentric, but oppose eccentric hypertrophy in vivo, regulating the balance between myocyte growth in width and length like ERK1/2 signaling^36^. Both ERK1/2 activation by expression of a constitutive MEK1 transgene and ERK1/2 (*Mapk1/Mapk3*) double knock-out resulted in cardiac hypertrophy; however, the former results in a non-fibrotic concentric hypertrophy with thickened myocytes^58^, while the latter resulted in ventricular dilation and elongated myocytes^36^. This model can explain the exaggerated eccentric hypertrophy in *Fgf21* targeted mice subjected to chronic isoproterenol infusion that promotes eccentric hypertrophy^26^. Likewise, the absence of fibrosis in wildtype mice given pharmacologic doses of FGF21 phenocopies MEK1 transgenic mice^58^.

Presumably via different downstream effectors that regulate myocyte apoptosis, MEK1 transgenic mice and ERK2 hemizygous knock-out mice exhibit decreased and increased infarct size, respectively, after ischemia–reperfusion^57^. Likewise, gain and loss of ERK1/2 signaling may explain the beneficial and deleterious effects on myocyte survival and infarct size of exogenous FGF21 and *Fgf21* knock-out, respectively, in mice subjected to ischemia–reperfusion injury or permanent coronary artery ligation^27,28^, although other ERK1/2-independent pathways may also be involved^31,33,34^. This pro-survival signaling could complicate the targeting of FGF21-FGFR4 signaling in diabetic cardiomyopathy. However, while *Fgf21* deletion increased myocyte apoptosis in streptozotocin-induced diabetic mice, it did not further worsen cardiac dysfunction^59^.

The identification of FGF21-FGFR4 signaling as a key regulator of concentric cardiac hypertrophy might provide a novel therapeutic opportunity to prevent or treat heart failure in diabetes. Targeting this pathway might complement the use of SGLT2 inhibitors that have recently been shown to be efficacious in clinical trials for diabetic cardiomyopathy and HFpEF^60,61^. While concentric cardiac hypertrophy can in theory reduce ventricular wall stress (Law of LaPlace), left ventricular hypertrophy is a major risk factor for future heart failure, such that extensive preclinical evidence suggests that preventing concentric hypertrophy even in the presence of persistently increased afterload (hypertension) would be ultimately beneficial^62^. In the context of diabetic cardiomyopathy, concentric hypertrophy predisposes the heart to significant interstitial fibrosis and diastolic dysfunction^63^. As shown here, inhibited FGFR4 signaling attenuated both cardiac hypertrophy and interstitial myocardial fibrosis in db/db mice. Similarly, inhibition of FGFR4 is a potential approach that might be useful for the cardiomyopathy associated with CKD^54^. Besides the potential use of biologic agents such as blocking antibodies, there are several recently developed small molecule FGFR4-specific inhibitors being considered for cancer indications in patients^64^. Alternatively, FGF21 neutralizing antibodies might be developed to inhibit FGF21 function in diabetic cardiomyopathy. Whether non-cardiac effects of FGF21-FGFR4 inhibition would be tolerated in the context of a chronic cardiovascular therapy will determine the potential of this approach. The lack of a severe phenotype in mice with global *Fgfr4* deletion is encouraging^65,66^. Regardless, readily reversible therapies would be desirable given the beneficial effects of FGF21 in ischemic heart disease, especially since those with diabetes are at risk for a coronary event.

Whether or not FGF21-FGFR4 signaling will prove to be a successful target for intervention against diabetic cardiomyopathy, the observation that FGF21 promotes concentric cardiac hypertrophy should be considered by the community actively developing FGF21 analogs and mimetics as agents to promote weight loss, hyperglycemia and dyslipidemia^8^. Besides the potential for bone loss, as well as the possibility that there may be “resistance” to the metabolic effects of elevated FGF21 in T2D^7^, further elevation in FGF21’s systemic activity may exacerbate the development of diabetic cardiomyopathy and other conditions characterized by concentric cardiac hypertrophy.

## Methods

### Antibodies and recombinant proteins

We used human FGF21 (2539-FG-025/CF, R&D Systems, United States), mouse FGF21 (8409-FG-025/CF, R&D Systems), human soluble β-klotho (5889-KB-050/CF, R&D Systems), human FGFR1c (658-FR-050, R&D Systems); human FGFR2c (712-FR-050, R&D Systems); human FGFR3c (766-FR-050, R&D Systems); and human FGFR4 (685-FR-050, R&D Systems). Anti-FGFR4 (human monoclonal, U3-11) was isolated by U3 Pharma/Daiichi-Sankyo (Germany) as described before^67^. This antibody is specific for FGFR4 and does not inhibit the activation of FGFR1-3^42^. We used a horseradish peroxidase-conjugated anti-human antibody (109035098, Jackson Immunolabs) for the plate-based binding assay. We used anti-ERK1/2 (9102 and 4695, Cell Signaling), anti-phospho-ERK1/2 (Thr202/Tyr204) (9101, Cell signaling) and anti-GAPDH (CB1001, EMD Millipore) for Western blot analyses.

### Isolation and analyses of adult rat ventricular myocytes

Eight-week-old male Sprague–Dawley rats were anesthetized with Ketamine (40–100 mg/kg i.p.) and Xylazine (5–13 mg/kg i.p.). Hearts were extracted and perfused using a Langendorff system with calcium depletion perfusion buffer containing 120 mM NaCl, 5.4 mM KCl, 1.2 mM NaH_2_PO_4_·7H_2_O, 20 mM NaHCO_3_, 1.6 mM MgCl_2_·6H_2_O, 5 mM taurine, and 5.6 mM glucose, 10 mM 2,3-butanedione monoxime (BDM), followed by type II collagenase digestion at 37 °C. The ventricular tissue was cut using sterilized scissors, and the cells were disassociated by pipetting and then filtered using 200 µm mesh. Ventricular myocytes (ARVMs) were collected by centrifugation at 50*g* for 1 min. ARVMs were then washed and purified using perfusion buffer containing 0.1% fatty acid-free BSA, 1 mM ATP (pH 7.2), 1 mM glutamine with CaCl_2_ added gradually until reaching a Ca^2+^ concentration of 1.8 mM. The purified ARVMs were then resuspended in a defined Minimal Medium [Medium 199 (11150059, Thermo Fisher, United States) supplemented with 5 mM creatine, 2 mM l-carnitine, 5 mM taurine, 25 mM HEPES, 0.2% fatty acid-free BSA, 10 mM BDM and insulin-transferrin-selenium (ITS)] and seeded onto square cover slips (21 mm × 21 mm) coated with mouse laminin (10 μg/mL diluted in PBS) in 6-well plates (10,0000 cells per well). One hour after isolation, the culture medium was removed, and ARVMs were washed with 2 mL medium once, then cultured for 48 h in Minimal Medium containing recombinant FGF21 (25 ng/mL, dissolved in 0.1% BSA) or 0.1% BSA control with and without phenylephrine (20 μM), isoproterenol (10 μM) or additional glucose (10 mM, final 15.6 mM) as indicated. To block FGFR4, isolated ARVMs were pre-treated with Minimal Medium containing FGFR4 blocking antibody (10 mg/mL human monoclonal U3-11; U3Pharma) for one hour before addition of FGF21 (25 ng/mL) and glucose (15.6 mM final). For morphometric analysis, ARVMs were washed twice with PBS and fixed with 3.7% formaldehyde solution (diluted in PBS) for one hour. Coverslips were mounted onto slides, and nine images per slide were obtained using a Leica DM4000 Microscope. The width and length of 100 ARVMs/image were measured by Leica LAX software. ARVM length and width were defined by the maximum dimensions of the cell either parallel and perpendicular to sarcomeric striations, respectively.

For Western blot analyses, ARVMs were washed with cold PBS and lysed in buffer (20 mM Tris–HCl, pH 7.6, 150 mM NaCl, 2 mM Na_2_EDTA, 1% NP-40, 1% sodium deoxycholate, 0.1% SDS, 2.5 mM sodium pyrophosphate, 1 mM beta-glycerophosphate, 1 mM Na_3_VO_4_, 5 mM NaF, and protease inhibitors). Protein concentrations were measured by Bio-Rad Protein Assay. Proteins were separated by SDS-PAGE electrophoresis. Precision Plus Protein marker was used to identify protein sizes. Equal loading was confirmed by Ponceau S total protein staining. Blots were incubated with primary antibody followed by donkey anti-IgG horseradish peroxidase antibody conjugates (Jackson Immune Research) secondary antibody. Super-Signal West Chemiluminescent Substrates (Thermo Scientific) and an Amersham Imager 600 Imaging System (GE Healthcare Life Sciences) were used to detect signals. ImageQuant™ TL software was used to quantify signals.

### Plate-based binding assay

To measure FGF21 binding to FGFRs, 96-well plates (Thermo Fisher; #44-2404-21) were coated with 200 ng of human FGF21 protein in 100 μL of coating buffer (E107, Bethyl) per well at 4 °C overnight. Plates were washed 5× with 350 μL of assay buffer (50 mM Tris pH 7.4, 200 mM NaCl, 0.01% Tween 20) on a 50TS microplate washer (BioTek, United States). Plates were blocked for 1 h in 200 μL assay buffer with 0.5% BSA (Rockland; BSA-50). Plates were washed 5× with assay buffer and incubated with 100 μL assay buffer with 0.5% BSA in combination with PBS or 1 μg of human soluble β-klotho at room temperature. After 1 h, plates were washed, and 500 ng of FGFR-Fc in 100 μL volume of assay buffer with 0.5% BSA were added per well. After 1 h at room temperature, plates were washed 5× in assay buffer and incubated with 100 μL anti-human Fc-HRP at 1:10,000. Plates were washed as above and 100 μL TMB substrate (E102, Bethyl) was added for 15–20 min until positive wells developed a dark blue color. Reactions were stopped with an ELISA stop solution (E115, Bethyl) and analyzed on a Synergy H1 plate reader (BioTek) at 450 nm wavelength. All samples were run in triplicates.

### RNA isolation and real-time PCR

RNA from total mouse hearts was extracted using Trizol (15596026, Invitrogen, United States). 2 μg total RNA was reverse transcribed using Qscript (95048, Quanta Biosciences, United States). Quantitative PCR reactions were carried out in the StepOne plus Real-Time PCR System (Applied Biosystems, United States) using FAST SYBR Green Master Mix (4385610, Applied Biosystems). Raw data was quantified via StepOneTM software v2.3 from life Technologies. Relative gene expression was normalized to expression levels of GAPDH and evaluated using the 2^−ΔΔCt^ method.

ARVM RNA was extracted using RNeasy mini kit (74104, Qiagen, United States) according to manufacturer’s instructions. 500 ng total RNA was reverse transcribed using a Reverse Transcription Kit (4368814, Applied Biosystems). Quantitative PCR reactions were performed using the QuantStudio 5 System (Thermo Scientific, United States) using Powerup SYBR Green Master Mix (A25742, Thermo Scientific). Raw data was analyzed using QuantStudio 5 qPCR Data Analysis Software (Thermo Scientific). Relative gene expression was normalized to expression levels of Hprt1 and evaluated using the 2^−ΔΔCt^ method. Primer sequences are presented in Table 1.

**Table 1:** Primer sequences.

Col5a1	Mouse	Forward	CTACATCCGTGCCCTGGT
		Reverse	CCAGCACCGTCTTCTGGTAG
Fibronectin	Mouse	Forward	AGACCATACCTGCCGAATGTAG
		Reverse	GAGAGCTTCCTGTCCTGTAGAG
Gapdh	Mouse	Forward	CCCATGTTTGTGATGGGTGT
		Reverse	GAGCTTCCCGTTCAGCTCT
Fgfr4	Mouse	Forward	TGGAGTCTCGGAAGTGCATC
		Reverse	TACACGGTCAAACAACGCCT
Hptr1	Rat	Forward	CCAGTCAACGGGGGACATAA
		Reverse	ATCCAACAAAGTCTGGCCTGT
c-Fos	Mouse	Forward	GCAGAAGGGGCAAAGTAGAGC
		Reverse	CTTCAAGTTGATCTGTCTCCGCTT
Egr-1	Mouse	Forward	ACCTGACCACAGAGTCCTTTTC
		Reverse	AGCGGCCAGTATAGGTGATG

### FGF21 transgenic mice

FGF21 transgenic mice expressing a mouse FGF21 cDNA under the control of the apolipoprotein (ApoE) promoter (C57BL/6-Tg(Apoe-Fgf21)1Sakl/J; The Jackson Laboratory Stock No: 021964) were as previously described^39^. Eleven hemizygous and 10 wild type mice, male and female, were analyzed by echocardiography at 4 and 6 months of age before euthanasia for further analysis. For molecular analysis of ERK1/2 signaling, RNA and protein was isolated from heart tissue of 8–12 week-old male mice. Expression levels of *c-Fos* and *Egr-1* were determined by qPCR and the activation/phosphorylation of ERK1/2 by immunoblotting.

For Western blot analyses, hearts were lysed in RIPA-based buffer (50 mM sodium phosphate pH 7.5, 200 mM NaCl, 1% Triton X-100, 0.25% deoxycholic acid) with addition of protease inhibitors (11836153001, Roche) and phosphatase inhibitors (P5726, P0044, Sigma-Aldrich) for 30 min. The lysate was centrifuged at 20,000*g* for 60 min to remove tissue debris. Samples were boiled in Laemmli sample buffer (1610737, Biorad) with 1.42 M 2-mercaptoethanol for 5 min. 20 μL of samples were loaded onto 12% SDS-PAGE gels and analyzed by Western blotting.

### FGF21 serial injections

FGF21 injections were conducted following a similar protocol as previously established for FGF23 by us^68^ and others^69,70^. Briefly, 12-week-old, male and female BALB/cJ mice (Stock No: 000651; The Jackson Laboratory, United States) underwent tail vein injections. The day before the experiment, mice underwent echocardiographic analysis, as described below. Mice were anesthetized using 2.5% isoflurane and placed on a heat pad. Mice were divided into 2 groups of 10, receiving either vehicle solution (isotonic saline) or mouse FGF21 protein. Per injection, we used 40 μg/kg of FGF21 dissolved in 200 μL of isotonic saline, with 8 h between injections for a total of 5 consecutive days. All injections were performed via the lateral tail vein. On the morning of the 6th day, 16 h after the final tail vein injection, animals underwent echocardiographic analysis and were sacrificed.

### ob/ob mice

ob/ob mice lacking leptin^43^ were studied in the genetic BTBR background (BTBR.Cg-*Lep*^*ob*^/WiscJ; Stock No: 004824; The Jackson Laboratory)^47^. At 3 months of age, 5 ob/ob and 5 wild type mice, male and female, were i.p. injected daily with PD173074 (P2499, Sigma Aldrich) at 1 mg/kg and 5 ob/ob and 4 wild type mice with saline for 6 weeks. Animals were sacrificed and samples prepared as described here.

### db/db mice

db/db mice which lack the leptin receptor^49^ were purchased from The Jackson Laboratory (B6.BKS(D)-*Lepr*^*db*^/J; Stock No: 000697). Five 4-week-old db/db mice were i.p. injected bi-weekly with 25 mg/kg of anti FGFR4 for 24 weeks. Eight db/db mice and 13 wild type mice were injected with vehicle as control. All groups contained male and female mice. Animals were monitored every 4 weeks for body weight and blood glucose levels and by echocardiographic measurements. After 24 weeks, animals were sacrificed, and samples were prepared as here.

### Histology and morphometry of mouse hearts

To prevent bias in the measurement of myocyte cross-sectional area using paraffin-embedded transverse cardiac sections, researchers taking images and measuring cell area were blinded. Heart tissue was washed with cold saline ex vivo and fixed overnight in 4% phosphate-buffered formaldehyde solution. Tissue was sent to IDEXX Bioanalytics (Columbia, MO) for embedding and sectioning. Transverse sections stained with hematoxylin and eosin (H&E) or following wheat-germ agglutinin (WGA) staining were imaged on a Leica DMi8 microscope. For WGA staining, paraffin sections underwent deparaffinization 2× for 5 min in Shandon Xylene Substitute and then rehydrated through a graded ethanol series (99%, 97%, 70%), 2× for 5 min each. Antigen retrieval was performed in a microwave for 15 min in 1× unmasking solution (H3300, Vector Labs). Slides were washed 3× for 5 min each in PBS, then incubated for 1 h in blocking solution ((1% BSA50 (Rockland), 0.1% cold water fish skin gelatin (900033, Aurion), and 0.1% Tween 20)). Slides were washed 3× in PBS and then incubated in 10 μg/mL of 594-conjugate WGA (W11262, Thermo Fisher) for 1 h. Slides were washed 3× with PBS and then mounted in Prolong Diamond (P36961, Thermo Fisher). Immunofluorescence images were taken with a 63× oil objective. ImageJ software (NIH) was used to quantify the cross-section area of 25 myocytes per field in 4 fields along the mid-chamber free wall based on WGA-positive staining.

### Echocardiography of mouse hearts

For mice receiving serial injections of recombinant FGF21 protein, transthoracic echocardiographic analysis was performed on day 6 of the experiment, at 13 weeks of age using a Vevo 770® High-Resolution Micro-Imaging System (FUJIFILM VisualSonics), equipped with an 707B-253 transducer. Animals were minimally anesthetized with 1–1.5% isoflurane, and normal body temperature was maintained using a rectal probe for temperature monitoring. For analysis, both B- and M-mode images were obtained in the short and long axis view. Correct positioning of the transducer was ensured using B-mode imaging in the long axis view, before switching to the short axis view. Parameters were calculated from at least three cardiac cycles by M-mode echocardiographs. All parameters were measured or calculated using Vevo® 770 Workstation Software (FUJIFILM VisualSonics).

Serial transthoracic echocardiography was performed in FGF21 Tg mice at the age of 16 and 24 weeks, and in db/db mice every 4 weeks from 4 weeks of age, using a Vevo 2100 High-Resolution Imaging System (FUJIFILM VisualSonics). Animals were minimally anesthetized with 1–1.5% isoflurane, and normal heart rate and body temperature were monitored and maintained throughout the procedure. Parameters were calculated from at least three cardiac cycles by M-mode echocardiographs. All parameters were measured or calculated using the VevoLAB software (FUJIFILM VisualSonics). Measurement definitions of short axis and calculations (M-Mode) are presented in Tables 2 and 3.

**Table 2:** Measurement definitions short axis (M-Mode).

Parameter	Definition	Unit
LVPW;d	Left ventricular posterior wall thickness (diastole)	mm
LVPW;s	Left ventricular posterior wall thickness (systole)	mm
LVAW;d	Left ventricular anterior wall thickness (diastole)	mm
LVAW;s	Left ventricular anterior wall thickness (systole)	mm
LVID;d	Left ventricular internal diameter (diastole)	mm
LVID;s	Left ventricular internal diameter (systole)	mm
Heart rate	Heart rate	BPM
Body weight	Body weight	g
LV mass/body weight	LV mass/body weight ratio	mg/g

**Table 3:** Calculations short-axis (M-Mode).

Parameter	Definition	Unit	Formula
LV Vol;d	Left ventricle volume in diastole (M-Mode)	µl	$\left(\frac{7.0}{2.4+LVID;d}\right) \times {LVID;d}^{3}$
LV Vol;s	Left ventricle volume in systole (M-Mode)	µl	$\left(\frac{7.0}{2.4+LVID;s}\right) \times {LVID;s}^{3}$
EF	LV ejection fraction (M-Mode)	%	$100 \times \left(\frac{LV Vol;d-LV Vol;s}{LV Vol;d}\right)$
FS	LV fractional shortening (M-Mode)	%	$100 \times \left(\frac{LVID;d-LVID;s}{LVID;d}\right)$
LV mass AW (Corrected)	LV mass AW corrected (M-Mode)	mg	$0.8\times \left(1.053\times \left({\left(LVID;d+LVPW;d+LVAW;d\right)}^{3}-{LVID;d}^{3}\right)\right)$
Concentricity	Relative wall thickness		$\left(\frac{LVAW;d+LVPW;d}{LVID;d}\right)$

### Serum chemistry

At endpoint, blood was collected from mice via cardiac puncture, transferred into microvette serum gel tubes (20.1344, Sarstedt) and centrifuged at 10,000*g* for 5 min. Serum supernatants were collected and stored at − 80 °C. ELISAs were run to detect FGF21 (MF2100, R&D Systems) and FGF23 (60-6300; Quidel).

### Statistics

Values are expressed as mean ± SEM if not otherwise indicated. Comparisons between 3 or more groups were performed by one-way ANOVA followed by post-hoc Tukey test or by 2-way ANOVA with post-hoc Sidak’s multiple comparison test. Comparisons between 2 groups were performed by two-tailed t-tests. A significance level of P ≤ 0.05 was accepted as statistically significant. No statistical method but experience from previous publications was used to predetermine sample size. No formal randomization was used in any experiment. For in vivo experiments, animals were unbiasedly assigned into different treatment groups. Group allocation was not performed in a blinded manner. Whenever possible, experimenters were blinded to the groups (for example, in IF and IHC experiments by hiding group designation and genotype of animals until after quantification and analysis).

### Study approval

All animal protocols and experimental procedures for FGF21 injections in mice, studying FGF21 Tg mice, and pan-FGFR and anti-FGFR4 injections in ob/ob and db/db mice were approved by the Institutional Animal Care and Use Committees (IACUC) at the University of Alabama Birmingham School of Medicine and the University of Miami Miller School of Medicine. The use of rats for myocyte isolation was approved by the Administrative Panel on Laboratory Animal Care at Stanford University. All animals were maintained in temperature-controlled environments with a 12-h light/dark cycle and allowed ad libitum access to food and water. All protocols adhered to the Guide for Care and Use of Laboratory Animals to minimize pain and suffering. All methods involving animals are reported in accordance with ARRIVE (Animal Research: Reporting of in Vivo Experiments) guidelines.

## Supplementary Information

Supplementary Information.
