# Supplementary material for: FGF21-FGFR4 signaling in cardiac myocytes promotes concentric cardiac hypertrophy in mouse models of diabetes
Source: Sci Rep. 2022 May 5;12:7326. doi: 10.1038/s41598-022-11033-x (PMC9072546; doi:10.1038/s41598-022-11033-x)
Supplement: Supplementary file 1 — Supplementary Information. [file 41598_2022_11033_MOESM1_ESM.pdf]

Supplementary Information

Supplementary Figure 1

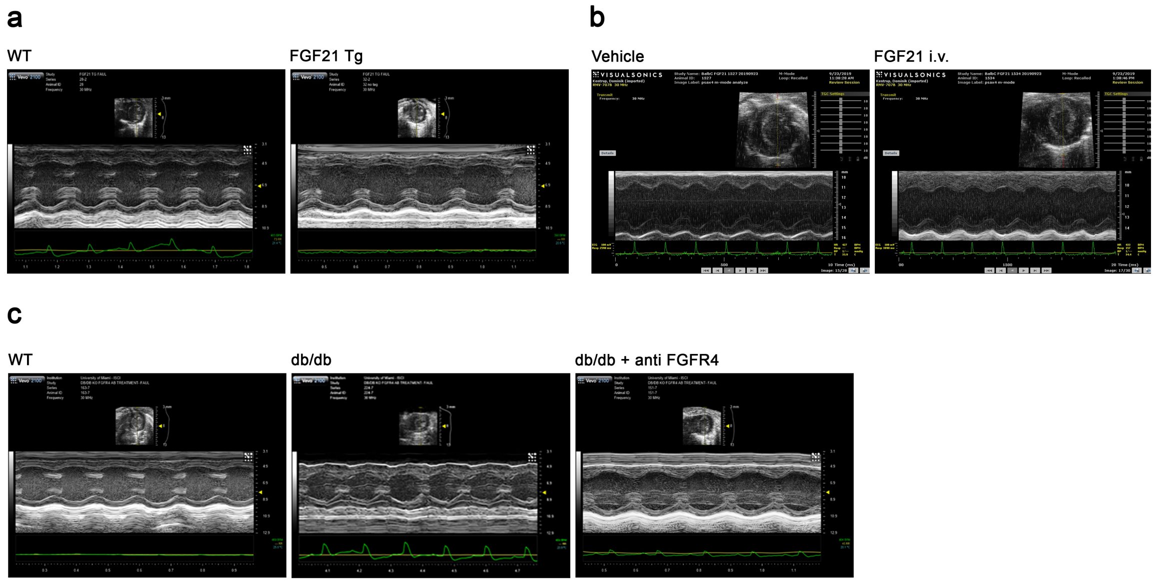

Supplementary Figure 1: Echocardiography M-mode images (short axis view)

Representative M-mode images of (a) FGF21 transgenic (Tg) mice and wild-type littermates at 24 weeks of age, (b) wildtype mice i.v. injected with either FGF21 or vehicle solution for five consecutive days, and (c) db/db mice and wild-type littermates at 28 weeks of age, after being treated for 24 weeks with either anti-FGFR4 (25 mg/kg) or vehicle solution on a bi-weekly basis, starting at 4 weeks of age.

Supplementary Figure 2

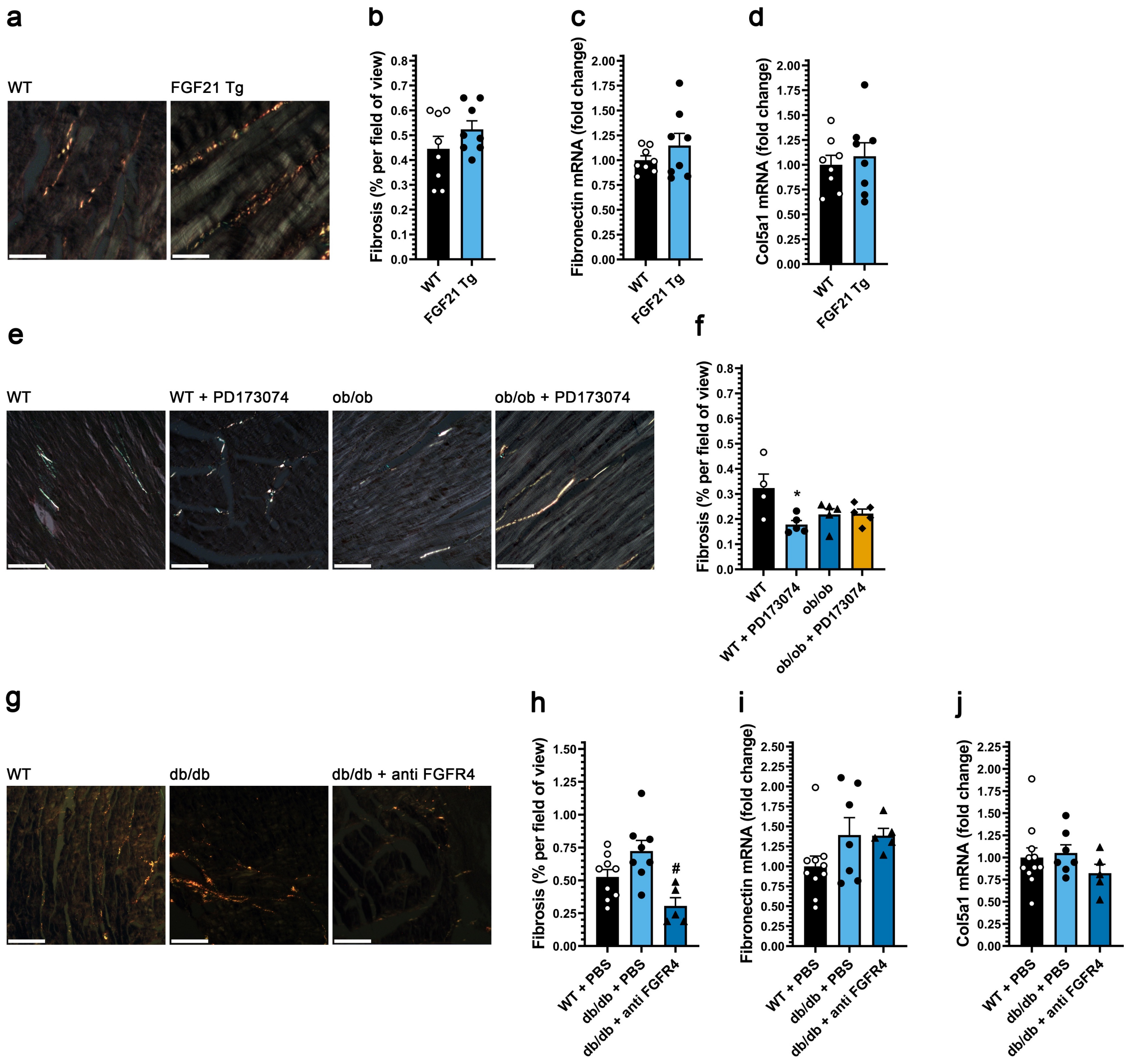

Supplementary Figure 2: Analysis of cardiac fibrosis

(a) Representative images of cardiac tissue sections of FGF21 transgenic (Tg) mice, stained with picrosirius red, taken under polarized light (scale bar = 50 μm), and (b) quantification of positive areas. Real-time qPCR analysis of fibrosis markers (c) fibronectin and (d) Col5a1 in cardiac tissue. (e) Representative images of cardiac tissue sections stained with picrosirius red, taken under polarized light (scale bar = 50 μm), of ob/ob mice and wild-

type littermates at three months of age, after being treated daily with 1 mg/kg PD173074 or vehicle solution for six weeks (scale bar = 2 mm), and (f) the quantification of stained area. (g) Representative images of cardiac tissue sections of db/db mice and wild-type littermates at 28 weeks of age, after being treated for 24 weeks with either anti-FGFR4 (25 mg/kg) or vehicle solution on a bi-weekly base, starting at 4 weeks of age, stained with picrosirius red, taken under polarized light (scale bar = 50 μm), and (h) the quantification of stained area. (i, j) Real-time qPCR analysis of fibrosis markers (i) fibronectin and (j) Col5a1 in cardiac tissue. Comparison between groups was performed in form of a one-way ANOVA (f, h-j) followed by a post-hoc Tukey test, or in form of a two-tailed t-test (b-d). A significance level of $p \leq 0.05$ was accepted as statistically significant. All values are expressed as mean ± SEM. b) N=8, *$p \leq 0.05$ vs. WT; c, d) N=8, *$p \leq 0.05$ vs. WT; f) N=4-5, *$p \leq 0.05$ vs. WT; h) N=5-9, *$p \leq 0.05$ vs. WT, #$p \leq 0.05$ vs. db/db; i, j) N=5-11, *$p \leq 0.05$ vs. WT, #$p \leq 0.05$ vs. db/db.

Supplementary Figure 3

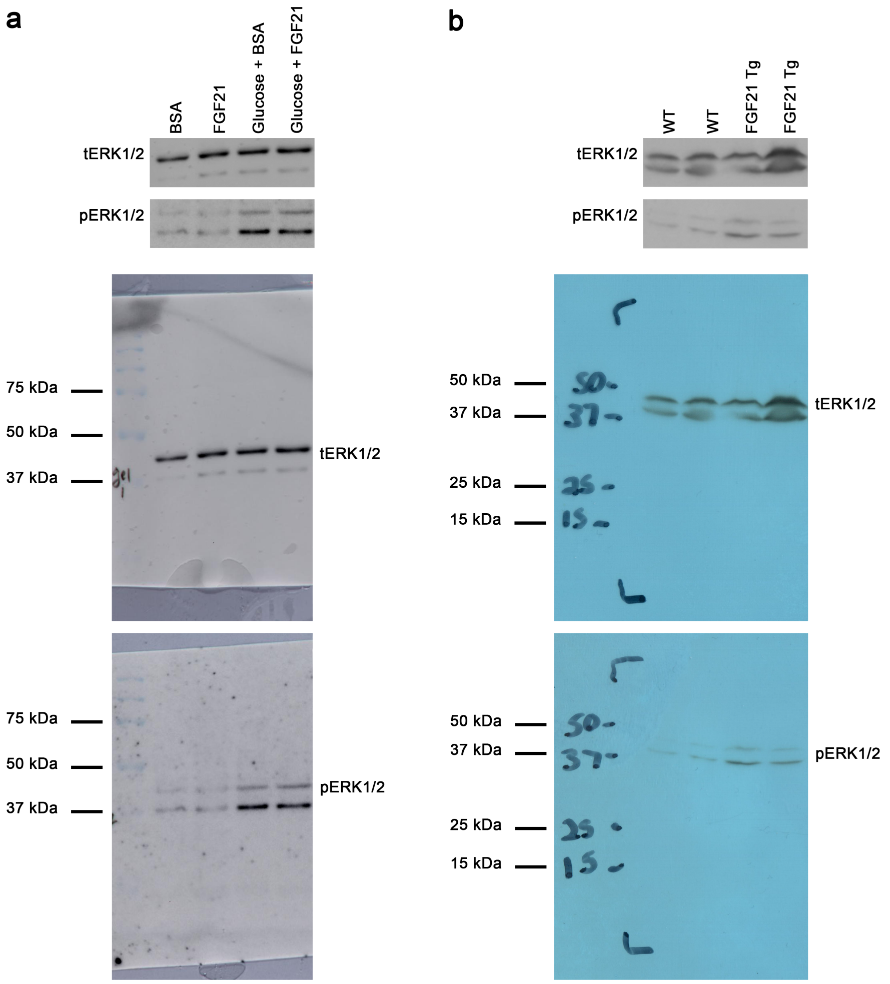

Supplementary Figure 3: Original Western blots

(a) Western blots as shown in Figure 3d, and original blots with molecular weight markers below. (b) Western blots as shown in Figure 3g, and original blots with molecular weight markers below.

Supplementary Table 1: Echocardiography of FGF21 Tg mice at 16 and 24 weeks of age and 24 week gravimetric data

		WT N=8			FGF21 Tg N=8			
**16 Weeks**								
LVPW;d	mm	0.71	±	0.06	0.62	±	0.08	*
LVPW;s	mm	0.97	±	0.09	0.97	±	0.21	
LVAW;d	mm	0.64	±	0.08	0.57	±	0.11	
LVAW;s	mm	0.95	±	0.13	0.92	±	0.15	
LVID;d	mm	4.07	±	0.24	3.38	±	0.26	***
LVID;s	mm	2.94	±	0.30	2.09	±	0.37	***
LV Volume;d	µL	75	±	10	48	±	7	***
LV Volume;s	µL	35	±	7	16	±	6	***
Ejection fraction	%	54	±	7	67	±	9	***
Fractional shortening	%	28	±	4	37	±	7	**
LV Mass	mg	85	±	13	49	±	8	***
Body weight	g	26	±	5	14	±	1	***
LV Mass/Body weight	mg/g	3.4	±	0.5	3.5	±	0.4	
Heart rate	BPM	438	±	30	434	±	32	
Concentricity		0.33	±	0.03	0.35	±	0.06	

		WT N=8			FGF21 Tg N=8			
**24 Weeks**								
LVPW;d	mm	0.74	±	0.06	0.76	±	0.05	
LVPW;s	mm	1.04	±	0.09	1.20	±	0.11	*
LVAW;d	mm	0.69	±	0.09	0.75	±	0.05	
LVAW;s	mm	1.03	±	0.11	1.17	±	0.07	
LVID;d	mm	3.98	±	0.25	3.38	±	0.26	***
LVID;s	mm	2.75	±	0.24	2.08	±	0.20	***
LV Volume;d	µL	70	±	10	50	±	8	***
LV Volume;s	µL	29	±	7	15	±	2	***
Ejection fraction	%	58	±	6	70	±	3	**
Fractional shortening	%	31	±	4	39	±	2	**
LV Mass	mg	81	±	11	67	±	10	**
Body weight	g	28	±	6	15	±	2	***
LV Mass/Body weight	mg/g	3.0	±	0.3	4.5	±	0.6	***
Heart rate	BPM	479	±	47	425	±	36	*
Concentricity		0.36	±	0.04	0.45	±	0.03	***
Heart weight	mg	124.3	±	5.5	78.0	±	3.1	***
Heart weight / Body weight (mg/g)	mg/g	4.7	±	0.2	5.4	±	0.1	***

Abbreviations: LVPW, Left ventricular posterior wall thickness; LVAW, left ventricular anterior wall thickness; LVID, Left ventricular internal diameter; LV, Left ventricular; d, diastole; s, systole; concentricity = (LVAW+LVPW)/LVID in diastole; BPM, beats per

minute. All data are mean ± SD. *$p \leq 0.05$, **$p<0.01$, ***$p<0.001$ vs. WT of same age. All p-values are based upon 2-way ANOVA Sidek post-hoc testing involving both datasets, or unpaired t-tests (Two-Tailed).

Supplementary Table 2: Echocardiography and gravimetric data of mice receiving serial FGF21 injections

		WT N=10			FGF21 i.v. N=10			
LVPW;d	mm	0.65	±	0.10	0.71	±	0.09	
LVPW;s	mm	1.17	±	0.10	1.17	±	0.09	
LVAW;d	mm	0.76	±	0.06	0.89	±	0.07	***
LVAW;s	mm	1.25	±	0.11	1.40	±	0.12	**
LVID;d	mm	3.78	±	0.39	3.61	±	0.27	
LVID;s	mm	2.31	±	0.43	2.11	±	0.35	
LV Volume;d	µL	62	±	15	55	±	10	
LV Volume;s	µL	19	±	9	15	±	6	
Ejection fraction	%	70	±	7	73	±	7	
Fractional shortening	%	39	±	5	42	±	6	
LV Mass (AW)	mg	73	±	15	80	±	15	
Body weight	g	25	±	3	25	±	5	
LV Mass (AW)/Body weight	mg/g	2.9	±	0.3	3.2	±	0.4	*
Heart rate	BPM	427	±	31	429	±	14	
Concentricity		0.38	±	0.04	0.44	±	0.03	**
Heart weight	mg	102.8	±	5.1	108.3	±	7.8	
Heart weight / Body weight (mg/g)	mg/g	4.0	±	0.1	4.3	±	0.1	**

Abbreviations: LVPW, Left ventricular posterior wall thickness; LVAW, left ventricular anterior wall thickness; LVID, Left ventricular internal diameter; LV, Left ventricular; d, diastole; s, systole; concentricity = (LVAW+LVPW)/LVID in diastole; BPM, beats per minute. All data are mean ± SD. *p≤ 0.05, **p<0.01, ***p<0.001 vs. WT + PBS. All p-values are based upon unpaired t-tests (Two-Tailed).

Supplementary Table 3: Echocardiography of db/db mice receiving anti-FGFR4 for 28 weeks

		WT + PBS<br>N=13			db/db + PBS<br>N=9				db/db + anti FGFR4<br>N=6			
**28 weeks**												
LVPW;d	mm	0.76	±	0.01	0.88	±	0.03	**	0.76	±	0.05	#
LVPW;s	mm	1.00	±	0.02	1.26	±	0.07	***	1.01	±	0.07	##
LVAW;d	mm	0.70	±	0.01	0.91	±	0.07	***	0.69	±	0.05	###
LVAW;s	mm	1.07	±	0.03	1.36	±	0.10	***	1.02	±	0.07	###
LVID;d	mm	4.05	±	0.12	4.11	±	0.17		4.28	±	0.13	
LVID;s	mm	2.85	±	0.14	2.76	±	0.22		2.95	±	0.22	
LV Volume;d	µL	76	±	5	76	±	7		84	±	5	
LV Volume;s	µL	34	±	4	32	±	6		35	±	5	
Ejection fraction	%	57	±	2	60	±	4		58	±	5	
Fractional shortening	%	30	±	2	32	±	3		31	±	3	
LV Mass	mg	87	±	4	114	±	6	**	93	±	7	
Body Weight	g	27	±	1	56	±	7	***	46	±	9	***
Heart rate	BPM	460	±	12	455	±	15		401	±	23	
Concentricity		0.36	±	0.04	0.45	±	0.11	***	0.34	±	0.07	###

Abbreviations: LVPW, Left ventricular posterior wall thickness; LVAW, left ventricular anterior wall thickness; LVID, Left ventricular internal diameter; LV, Left ventricular; d, diastole; s, systole; concentricity = (LVAW+LVPW)/LVID in diastole; BPM, beats per minute. All data are mean ± SD. *p≤ 0.05, **p<0.01, ***p<0.001 vs. WT + PBS of same age, #p≤ 0.05, ##p<0.01, ###p<0.001 vs. db/db + PBS of same age. All p-values are based upon 2-way ANOVA and Tukey post-hoc testing involving full-time course dataset.
